# Genome-Wide Analysis of the AT-Hook Gene Family in *Malus sieversii* and Functional Characterization of MsAHL13

**DOI:** 10.3390/plants14172625

**Published:** 2025-08-23

**Authors:** Da Zhang, Chao Zhao, Xin Liu, Han Wang, Bowei Zhu, Guodong Zhao, Dongmei Chen, Tongsheng Zhao, Haijiao Xu, Yingjie Wang, Chaohong Zhang, Xinsheng Zhang

**Affiliations:** 1 Changli Institute of Pomology, Hebei Academy of Agriculture and Forestry Sciences, Qinhuangdao 066600, China; d.zhang@nwafu.edu.cn (D.Z.); 18830180711@163.com (X.L.); 15830298656@163.com (H.W.); 18331561549@163.com (B.Z.); guodong19823@163.com (G.Z.); chendm2009@126.com (D.C.); tshzh71@163.com (T.Z.); xuhaijiao1234@sina.cn (H.X.); 17733503931@163.com (Y.W.); 2 Shijiazhuang Institute of Pomology, Hebei Academy of Agriculture and Forestry Sciences, Shijiazhuang 050000, China; zhaochao123@nwafu.edu.cn

**Keywords:** *Malus sieversii*, MsAHL13, gene family, biotic and abiotic stresses

## Abstract

AT-hook motif nuclear-localized (AHL) proteins are pivotal in plant growth, development, and stress responses. Nevertheless, there is limited research on AHL proteins in *Malus sieversii*. Our study identified 25 *AHL* genes from the *M. sieversii* genome, named MsAHL1–MsAHL25. The encoded protein sequences had lengths ranging from 195 to 554 amino acids, molecular weights from 19.17 to 58.53 kDa, and isoelectric points from 4.67 to 10.09. Chromosomal mapping revealed that these 25 genes were unevenly distributed across 10 chromosomes. Collinearity analysis of *AHL* genes in *M. sieversii* implied that gene loss might have occurred during its evolution. The phylogenetic tree classified the AHL proteins of *M. sieversii* into two subfamilies, showing a close relationship with multiple proteins of *M. domestica*. Promoter analysis indicated that the *AHL* genes in *M. sieversii* harbored numerous stress- and hormone-responsive elements, suggesting their potential role in various stress responses. qRT-PCR analysis of six representative *MsAHLs* under biotic and abiotic stresses demonstrated that the expression of MsAHL13, MsAHL15, and MsAHL17 was significantly upregulated under salt, drought, and cold stresses, while MsAHL01 expression was inhibited under low-temperature stress. All six *MsAHLs* were induced by the pathogen *Valsa mali*. Subcellular localization analysis of the specifically expressed protein MsAHL13 showed its nuclear location. Furthermore, luciferase and yeast two-hybrid assays confirmed the in vitro physical interaction between the MsAHL13 and MsMYB1 proteins. This research offers an important theoretical basis for further exploration of the functional mechanisms of this gene family in responding to environmental stresses.

## 1. Introduction

The AT-hook motif nuclear-localized (AHL) family is a conserved transcription factor family. The AHL gene family is widely distributed among sequenced plants [1], such as *Arabidopsis thaliana* [2], rice (*Oryza sativa*) [3], tomato (*Solanum lycopersicum*) [4], maize (*Zea mays*) [5], poplar (*Populus trichocarpa*) [6], and walnut (*Juglans regia* L.) [7]. AHL proteins contain two conserved domains, the uncharacterized plants and prokaryote conserved (PPC, DUF296) domain and the AT-hook motif [8]. PPC, which contains about 120 amino acids, is responsible for their nuclear localization and interaction with other proteins [9]. Upstream of the Gly-Arg-Phe-Glu-Ile-Leu sequence, the type A PPC domain contains a core conserved sequence, Leu-Arg-Ser-His, while the type B PPC domain contains a core conserved sequence, Phe-Thr-Pro-His. The AT-hook motif is a small DNA-binding protein motif [3,10]. The AT-hook motif is characterized by a conserved core sequence, Arg-Gly-Arg-Pro, which enables it to specifically bind to the minor groove of AT-rich DNA regions [1,11]. The AT-hook motif can be divided into two types based on downstream amino acid sequences, characterized by the conserved sequences Gly-Ser-LysAsn-Lys (GSKNK) and Arg-Ly-Tyr (RKY), respectively [12].

The AHL gene family plays a crucial role in plant growth, development, and stress resistance. *AHL* genes are involved in regulating target gene expression and interacting with other proteins [1,8,12]. The *Arabidopsis* genome contains 29 *AHL* genes [2]. Research on *Arabidopsis thaliana* has revealed that the *AHL* gene family plays a role in regulating root growth and development, floral organ formation and development, and leaf growth and development [8,13,14,15]. Moreover, it is involved in modulating the balance of hormones such as abscisic acid (ABA) [16], gibberellin (GA) [17], jasmonic acid (JA) [18], and cytokinin (CK) [19]. The *Arabidopsis thaliana AHL* gene family redundantly regulates hypocotyl growth through PPC/DUF296 domain-mediated intermolecular interactions. AtAHL18 modulates root system architecture by regulating the activity of the root apical meristem. AtAHL3 and AtAHL4 regulate vascular tissue boundaries in the *Arabidopsis* root meristem [20]. *AHLs* repress petiole growth by antagonizing PIF-mediated transcriptional activation of genes associated with growth and hormone pathways. The *DP1* gene influences palea formation and floral organ development in rice [9]. In walnut (*J. regia* L.) research, it was found that overexpression of *JrAHL2* results in the inhibition of hypocotyl elongation and a delay in flowering in *Arabidopsis* [7]. *OsAHL* genes may be involved in mediating salt stress and drought signal transduction pathways in rice [3]. Additionally, overexpression of *OsAHL1* significantly enhances drought resistance in rice plants [3]. OsAHL1 regulates root development under drought conditions to enhance drought avoidance, and participates in the oxidative stress response. In *Brassica rapa*, *BrAHL16*, *BrAHL2*, and *BrAHL24* play critical roles in cold tolerance [21]. The *VvAHL* gene is involved in stress response and hormone signaling pathways in grape (*Vitis vinifera*) [22]. In maize (Zea mays), researchers discovered that the AT-hook motif nuclear-localized (AHL) transcription factor ZmAHL25 binds to the AT-rich cis-element in the *ZmPUB19* promoter and activates its expression upon *Rhizoctonia solani* infection, thereby enhancing disease resistance in maize. [23]. The chili pepper (*Capsicum annuum* L.) CaATL1 enhances disease resistance against bacterial [24]. In Poplar (*Populus trichocarpa*), PtrAHL34 induced by drought stress in roots and leaves can enhance drought tolerance. *M. sieversii* is widely recognized by the public for its exceptional environmental adaptability [25]. While previous studies have reported the identification of the *AHL* gene family in the *Malus domestica* [26], no such research on the AHL family in *M. sieversii* has been documented to date.

The objective of this study is to identify and analyze the *AHL* gene family in *M. sieversii* at a genome-wide level. According to the findings of the *AHL* gene family in *Arabidopsis thaliana*, a total of 29 genes were identified based on high-quality apple genome data [2]. The gene structure, conserved motif, subcellular localization chromosome locations, phylogenetic analysis, synteny analysis, promoter cis-elements, and protein interaction network of 25 AHLs found in apple were all investigated. Moreover, through qRT-PCR analysis of six selected *MsAHLs*, we found that these six genes responded to multiple stress environments to varying degrees. Finally, we also verified the subcellular localization and interacting proteins of MsAHL13. The implementation of this study aims to provide new ideas for apple breeding.

## 2. Result

### 2.1. Identification and Phylogenetic Analysis of Members of the AHL Gene Family in M. sieversii

**Figure 1:**
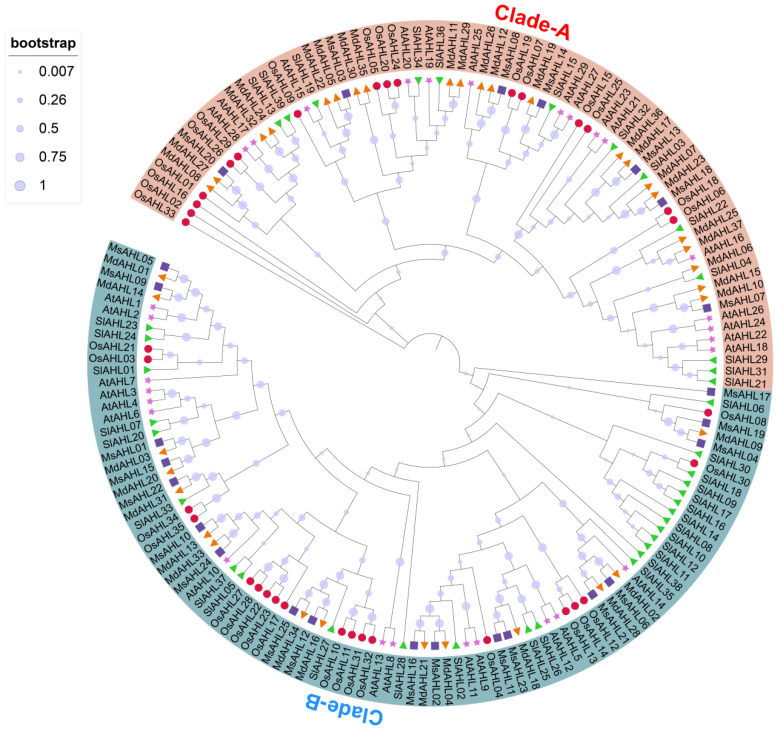
Systematic evolutionary analysis of AHL family proteins. Red circle, *Oryza sativa*; Yellow triangle, *Malus domestica*; Green triangle, *Solanum lycopersicum*; Pink pentagram, *Arabidopsis thaliana*; Purple square, *Malus sieversii*.

Using Pfam and SMART, we identified protein sequence domains for 29 candidate genes, excluding those without the RGRP motif. Ultimately, 25 apple AHL protein sequences were identified from the *M. sieversii* genome (Table S1). Using the ProtParam tool, we analyzed the physicochemical properties of the proteins encoded by these 25 genes (Table S1), revealing significant differences among various AHL protein sequences. The amino acid length ranged from 195 to 554 aa, with most sequences falling between 310 and 380 aa. Protein molecular weight ranged from 19.17 to 58.53 kDa. Isoelectric points varied from 4.67 to 10.09, with seven proteins having an isoelectric point below 7, indicating acidity, while the rest had an isoelectric point above 7, indicating alkalinity. Most apple AHL protein instability indices were greater than 40, except for MsAHL19 (39.49). The average hydrophilicity of the proteins ranged from −0.690 to 0.043. Predicting their functions in the apple genome database revealed that most apple AHL proteins, in addition to containing the conserved RGRP motif, also have PPC domains. Subcellular localization predictions showed that all AHL genes are located in the nucleus. Additionally, we constructed a phylogenetic tree through multiple sequence alignment for 37 *Malus domestica* var. ‘Golden Delicious’, 29 *Arabidopsis*, and 35 rice AHL proteins, as well as 39 tomato sequences (Figure 1). The results indicated significant differences in clustering between *M. sieversii* and apple AHL family proteins. In Clade-A, there were eighteen apple AHL proteins compared to only five *M. sieversii* proteins, suggesting that apple AHL proteins have evolved more functionally similar AHL proteins relative to *M. sieversii*. Furthermore, 10 *MsAHL* members clustered with 15 *Arabidopsis* AHL Clade-B subfamily members, which can be classified into Subgroup I. Nine *M. sieversii* *MsAHL* members clustered with fourteen *Arabidopsis* AHL proteins (Clade-A), showing closer kinship and belonging to Subgroup II. Overall, *M. sieversii* proteins exhibit more conservative evolution.

### 2.2. Analysis of MsAHL Gene Structure, Motifs, and Domains

**Figure 2:**
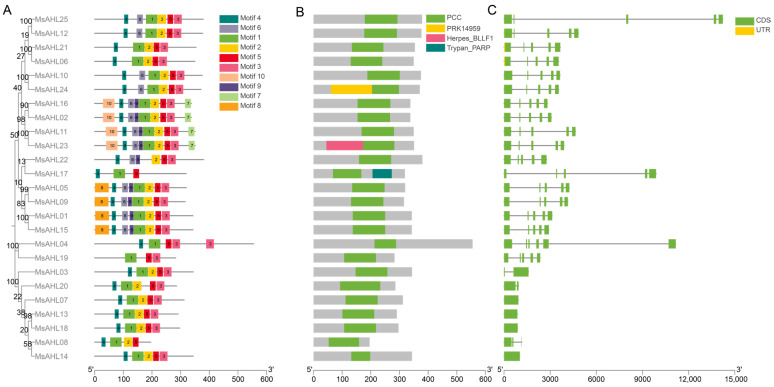
Analysis of conserved motifs, domains, and gene structures of the *MsAHLs*. (**A**). Phylogenetic tree and conserved motif analysis of the *MsAHLs*. (**B**). Conserved domain analysis of *MsAHLs*. (**C**). Gene structure analysis of *MsAHLs*.

We conducted a gene structure analysis of the *MsAHLs* in *M. sieversii* (Figure 2) and found that most members of the *MsAHL* gene family have a relatively stable structure, containing one to four exons and one to three introns, with most genes having two exons and one intron. MsAHL03/13/14/18 only contain one exon and no introns. Additionally, only MsAHL06 has UTR sequences (Figure 2B). Subsequently, we analyzed the conserved motifs of the AHL proteins in *M. sieversii* and discovered that the 25 conserved motifs range from 15 to 50 amino acids in length. As shown in Figure 2, all AHL proteins in *M. sieversii* contain 3 to 10 conserved motifs. Among them, MsAHL02/11/16/23 have the most conserved motifs, totaling nine (Motif 1, Motif 2, Motif 3, Motif 4, Motif 6, Motif 7, Motif 9, Motif 10); MsAHL17 has the fewest conserved motifs, totaling three (Motif 1, Motif 4, Motif 5). From the phylogenetic tree results in Figure 2A, it can be seen that members with close evolutionary relationships usually possess the same conserved motifs. It was also observed that most AHL proteins in *M. sieversii* contain the Motif 1 and Motif 5 conserved motifs. Overall, the AHL proteins in *M. sieversii* exhibit high conservation. The analysis of the conserved domains of the *M. sieversii* AHL proteins is shown in Figure 2B, where all AHL proteins contain a conserved PPC domain; among them, MsAHL17, MsAHL23, and MsAHL24, in addition to the PPC domain, also contain other domains.

### 2.3. Chromosome Mapping of MsAHLs

The location information of the *M. sieversii MsAHLs* was obtained from the apple genome database, and the chromosome map of the gene was drawn using TBtools software(V2.056) (Figure S1). From the figure, it can be seen that the apple *AHL* genes are unevenly distributed across the 10 chromosomes of the *M. sieversii* with no genes located on 7 of the chromosomes. The sixth chromosome has the highest distribution, containing five genes, followed by the first, fourth, and seventh chromosomes, which contain three, three, and four *MsAHLs*, respectively. In summary, the apple *MsAHL* gene family shows a single-copy distribution in the genome.

### 2.4. Analysis of Cis-Acting Elements

**Figure 3:**
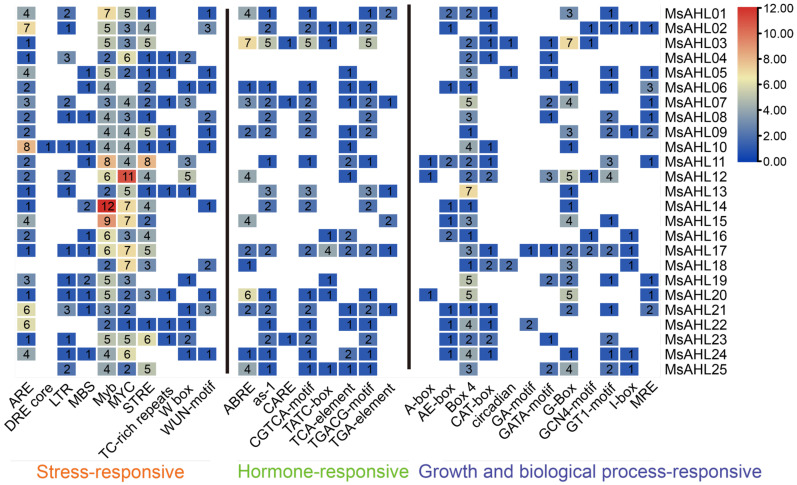
Analysis of cis-acting elements of the *MsAHLs*.

**Figure 4:**
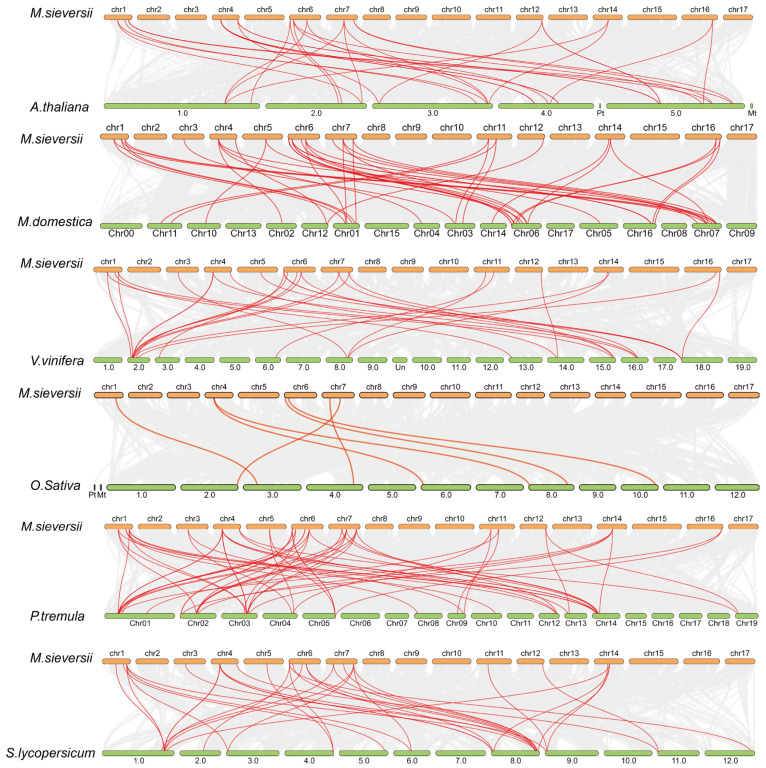
Collinearity analysis of the *MsAHL* family genes of *M. sieversii*. The red lines indicate syntenic relationships between *MsAHL* family genes and homologous genes in other species. The gray lines represent all syntenic gene pairs between the *M.sieversii* genome and the genomes of other species.

**Figure 5:**
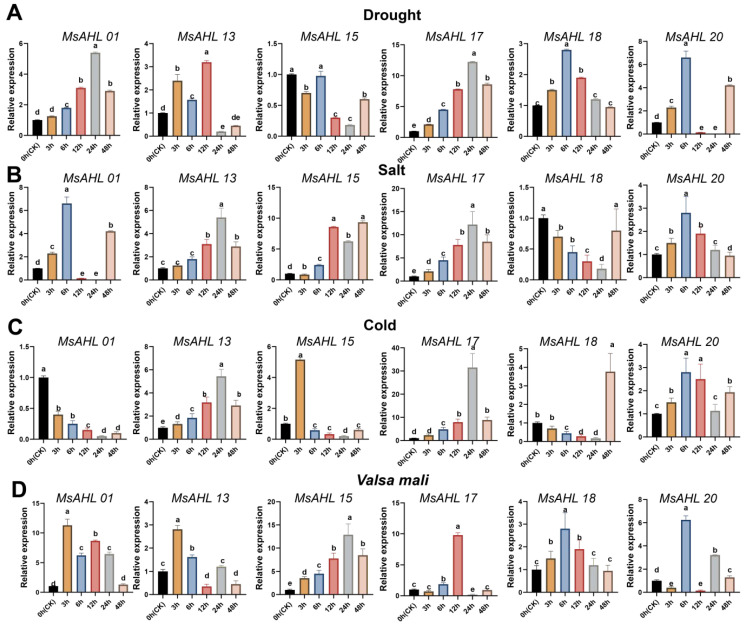
Quantitative expression of six *MsAHLs* genes under biotic and abiotic stresses. (**A**) Response of *MsAHLs* to drought stress. (**B**) Response of *MsAHLs* to salt stress. (**C**) Response of *MsAHLs* to cold stress. (**D**) Response of *MsAHLs* to *Valsa mali*. Apple seedlings were treated with PEG6000, NaCl, low temperature (4 °C), and *Valsa mali,* respectively. Samples were harvested at the indicated time points. Color-coded bars depict gene expression profiles in untreated controls versus various treatment time points. Results were normalized against the expression of *Actin* as an internal control. Values are means ± SD (*n* = 3). Different letters above the columns indicate significant differences (*p* ≤ 0.05). CK, control.

The promoter analysis of the 2 kb upstream fragments of the members of the *MsAHL* gene family in *M. sieversii* was carried out using PlantCARE (Figure 3). The results showed that there were 31 types of cis-acting elements in the promoter regions of 25 *MsAHLs*, mainly including light-responsive elements (45%), plant hormone-responsive elements (32%), abiotic stress-responsive elements (23%), and development-related elements. Among them, light-responsive elements accounted for the largest proportion, and plant hormone-responsive elements (such as those for ethylene, GA, Auxin, MeJA, and ABA) were widely distributed (Figure 4). Secondly, among the stress-responsive elements, the drought-responsive elements (such as MYB) and low-temperature-responsive elements (such as MBS) were the most numerous, accounting for 45% and 20% of the total abiotic stress-responsive elements, respectively (Figure 5). There were differences in the composition of cis-acting elements among *MsAHLs*, but the differences in the composition of cis-acting elements between the two sub-families were not significant. *MsAHL03* and *MsAHL13* contained more than three jasmonate-responsive elements (TGACG-motif and CGTCA-motif), with *MsAHL03* having the most (five), while the other genes had none or less than one jasmonate-responsive element. *MsAHL13* contained 12 drought-inducible elements (MYB), which were present in all genes. All *MsAHLs* except *MsAHL19*, *MsAHL21*, and *MsAHL24* contained at least one abscisic acid-responsive element (STRE). Compared with other members, the promoter region of *MsAHL20* had 7–13 more light-responsive elements than other genes, with the largest number of G-Boxes. *MsAHL12* had the most stress-responsive elements (21). Among the plant hormone-responsive elements, the abscisic acid-responsive elements were the most numerous, and *MsAHL12* and *MsAHL13* had the most plant hormone-responsive elements.

### 2.5. Collinearity Analysis of MsAHLs

To explore the evolution of 25 *MsAHL* family genes in *M. sieversii*, we conducted synteny analysis (Figure 4). Initially, we performed synteny analysis on model species such as *Arabidopsis*, rice, and tomato. We found that the number of syntenic gene pairs with rice was the lowest, with only 7 pairs, while the number with tomato was the highest, reaching 30 pairs. This suggests that, in model plants, the *MsAHL* family genes evolved more towards tomatoes, and there are more AHL family proteins in tomatoes. During the evolution to *M. sieversii*, some AHL proteins may have been lost. Additionally, we also conducted synteny analysis between closely related species. We discovered that when analyzing with cultivated *Malus domestica* ‘Golden Delicious’, grape, and poplar, *M. sieversii* had the most syntenic gene pairs with ‘Golden Delicious’. The numbers in grape and poplar were relatively similar. This indicates that, within the same species, *MsAHL* family genes have undergone more conserved evolution during their development, resulting in functional conservation.

### 2.6. Expression Analysis of Six MsAHLs

The expression patterns of six *MsAHLs* in *M. sieversii* under abiotic stresses will be further confirmed (Figure 5). The results showed that, under PEG6000 (15%) simulated drought stress (Figure 5A), the expression of *MsAHLs* in *M. sieversii* exhibited a trend of first increasing and then decreasing, except for *MsAHL15*. At 12 h, the expression levels of *MsAHL01*, *MsAHL13*, *MsAHL17*, and *MsAHL18* were significantly upregulated compared to the control, while *MsAHL20* was upregulated at 3, 6, and 48 h, and downregulated at 12 h. Additionally, at 24 h, the expression levels of *MsAHL13* and *MsAHL15* were downregulated, while the rest were upregulated. Compared to the control, *MsAHL20* was upregulated at 3, 6, and 48 h, and significantly downregulated at other time points. Under NaCl simulated salt stress (Figure 5B), all genes were induced to high expression to varying degrees, except for *MsAHL18*. Compared to the control, *MsAHL15* was significantly upregulated at all time points, with increases of 18.71%, 78.65%, 48.74%, 63.09%, and 143.71% (*p* < 0.05). *MsAHL18* was significantly downregulated at 3, 6, 12, and 24 h, with decreases of 10.85%, 26.11%, 38.12%, and 68.13%, respectively. *MsAHL20* reached its highest expression at 6 h, 99.19% higher than the control. Under 4 °C simulated cold stress (Figure 5C), *MsAHL01* was significantly inhibited at all five time points, while the rest of the genes were induced to varying degrees. Notably, *MsAHL15* responded more actively, showing significant upregulation at the third hour of treatment. Finally, we used *Valsa mali* to simulate fungal infection (Figure 5D) and observed that only *MsAHL01*, *MsAHL13*, and *MsAHL18* responded more actively, with significant upregulation at the 3h of infection. In conclusion, through the expression analysis of six representative *MsAHLs* in *M. sieversii*, we found that they responded to different stress treatments to varying degrees.

### 2.7. MsAHL13 Is Localized to the Nucleus

**Figure 6:**
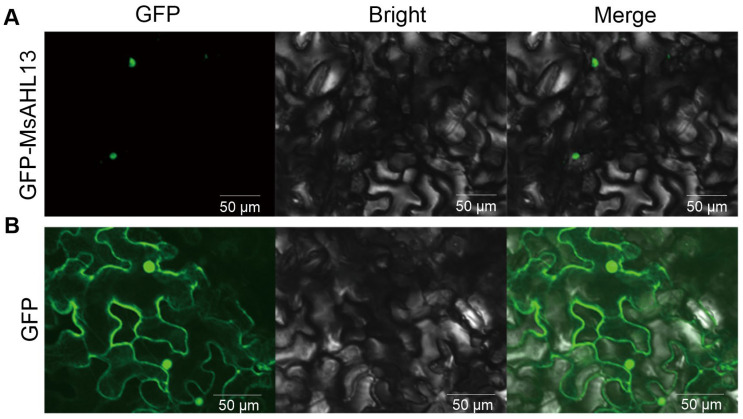
Subcellular localization of MsAHL13 in *Nicotiana benthamiana* leaves. (**A**) Confocal microscopy image of *35S:GFP*-*MsAHL13* fusion protein. (**B**), Control image of empty *35S:GFP* vector under identical confocal microscopy conditions. GFP, green fluorescent protein. Bright, bright-field. Merge, merge of bright-field and GFP images. Bars = 50 µm.

Given the distinct expression patterns observed under stress treatments, *MsAHL13* was identified as a candidate for subsequent characterization. *Agrobacterium*-mediated transient expression of pMCAMBIA1302-MsAHL13-GFP and the pMCAMBIA1302-GFP empty vector was performed in tobacco leaves. Observation under a laser confocal microscope (Figure 6) revealed that the green fluorescence of the *pCAMBIA1302-GFP* empty vector was distributed throughout the entire cell (Figure 6B), whereas the green fluorescence of the *pCAMBIA1302*-*MsAHL13* fusion protein expression vector was specifically localized in the nucleus, indicating that MsAHL13 is localized in the nucleus (Figure 6A).

### 2.8. Physical Interaction Between MsAHL13 and MsMYB1

**Figure 7:**
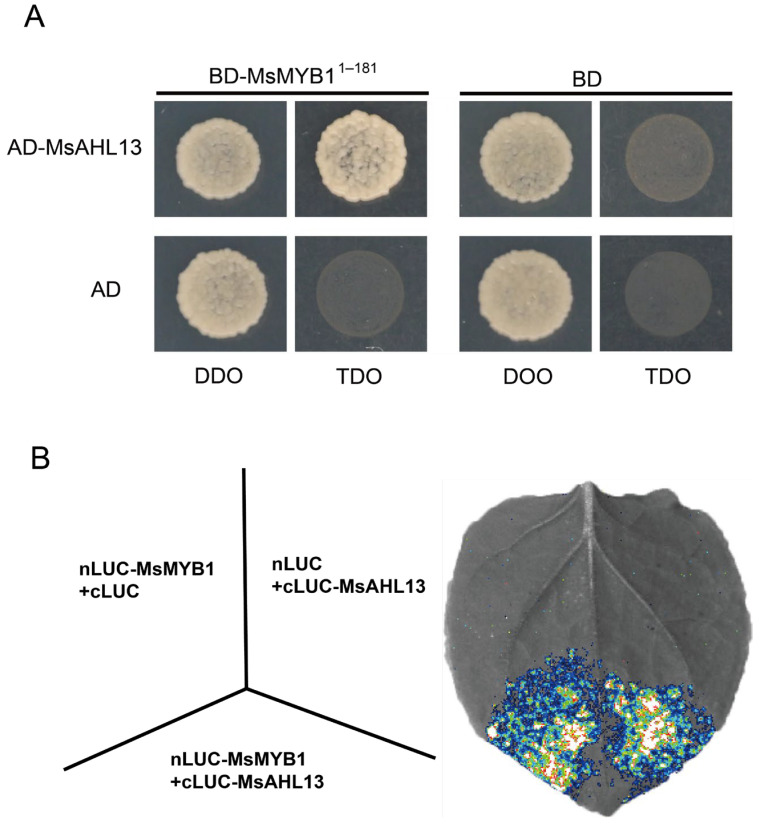
The validation of the protein interaction between MsAHL13 and MsMYB1. (**A**) Yeast two-hybrid assay. The empty GAL4 activation domain (AD) and GAL4 DNA-binding domain (BD) vectors were used as negative controls. TDO and DDO refer to yeast deficient-type medium; DTO, SD/-Trp/-Leu. TDO, SD/-Trp/-Leu/-His. (**B**) Luciferase (LUC) assay. The empty nLUC and cLUC vectors were used as negative controls. nLUC, luciferase N-terminal halves. cLUC, luciferase C-terminal halves.

The biosynthesis of anthocyanin is mediated by multiple abiotic and biotic stress signals [27]. To investigate whether MsAHL13 participates in the MsMYB1-mediated anthocyanin regulatory pathway, the coding sequence of the candidate interacting protein *MsMYB1* was cloned into the pGBKT7 vector to construct the pGBKT7-MsMYB1 vector (Figure 7). Similarly, the pGADT7-*MsAHL13* fusion expression vector was constructed. Both the pGADT7-*MsAHL13* and pGBKT7-*MsMYB1* plasmids were co-transformed into yeast AH109, and the interaction between MsAHL13 and MsMYB1 was detected on SD/-Leu-/Trp/-His medium. Results showed that yeast co-transformed with pGADT7-*MsAHL13* and pGBKT7-*MsMYB1* grew on SD/-Leu/-Trp/-His medium (Figure 7A). The result indicates that the candidate interacting protein MsMYB1 interacts with MsAHL13 in yeast. To further validate their interaction, MsAHL13 and MsMYB1 were separately constructed into pCAMBIA1300-cLUC and pCAMBIA1300-nLUC vectors (Figure 7B). After inoculation into tobacco plants, significant fluorescence was observed at the injection sites co-inoculated with nLUC-*MsMYB1* and cLUC-*MsAHL13* (Figure 7B). In conclusion, we deduce that MsMYB1 physically interacts with MsAHL13.

## 3. Discussion

As a unique and conserved transcription factor, AT-hook was first described in mosses and flowering plants and is highly conserved in all land plants [1,14]. AHLs play an important role in the transcriptional regulation of land plants, and their transcriptional regulation is mainly localized in the nucleus [27,28,29]. It has been proven that some conserved transcription factor families are essential for plant growth and stress tolerance during plant evolution, including the bHLH and NAC gene families [30,31,32,33]. However, some transcription factor families that play important roles in plant evolution have not been fully studied. It is necessary to understand the mechanism of action and function of AT-hook transcription factors at the transcriptional level. In this study, a bioinformatic analysis was conducted on 25 *MsAHLs*, which is 11 fewer than the number reported in the *Malus domestica* cultivar ‘Golden Delicious’ previously [26]. This indicates that the different growth environments of the cultivated variety ‘Golden Delicious’ and *M. sieversii* may lead to different functions of *MsAHLs*, resulting in the difference in their numbers. The chromosomal distribution of *MsAHLs* is concentrated at both ends, and such a distribution may be related to their biological functions (Figure 3).

Phylogenetic analysis shows that AHLs proteins in land plants first appeared in embryophytes and evolved into two different branches, namely, group A and B (Figure 1), which occurred earlier than the differentiation of bryophytes or other land plants. Researchers further classified AHLs into two types (Type-I/Type-I-II/Type-I-III) based on the functional domains of AHLs [3,4], the number and composition of AT-hook motifs, and PPC domains. This classification is also applicable to wild *M. sieversii* and is consistent with the previous classifications in other land plants [5,21]. It is also consistent with the previous classification of AHLs in apples.

Gene duplication mainly includes three forms: segmental duplication, random duplication, and reverse transcription [34]. Polyploidization in plants has retained a large number of replicated chromosomal segments in the genome, and gene replication caused by chromosomal segment replication is the most common in the genome [35]. Our collinearity analysis found that the genes encoding AT-hook proteins in the genome of *M. sieversii* may have been lost or expanded during the evolutionary process.

Conserved motif analysis showed that all 25 *AHL* genes of *M. sieversii* contained the conserved motif Motif 1. In addition, through the analysis of conserved structures, it was found that Motif 1 was included in the conserved domain of the AHL gene family, indicating that Motif 1 is a highly conserved motif in this gene family, which is conducive to the specific functions of AHL proteins, such as maintaining genome stability, DNA methylation, replication, and transcriptional regulation [36,37]. The structure of *AHL* genes in *M. sieversii* is relatively simple, with 25 members having only one exon and no introns, which is highly similar to the gene structure of *AHL* genes in species such as rice [3], corn [5], cotton [38], and radish [39]. Genes with no introns or only one intron cannot achieve the translation of a single gene into multiple proteins through alternative splicing, and their functions tend to be more conservative and singular in evolution. This indicates that the AHL gene family is highly conserved.

Cis-acting elements are binding sites for transcription factors. Plants can adapt to environmental stress by regulating transcription rates [40,41]. The activation or inhibition of transcription of stress-related genes plays a key role in the regulation of plant abiotic stress [42,43]. In *M. sieversii*, 12 *MsAHLs* contain response elements related to low temperature, and 16 *MsAHLs* have binding sites for MYB transcription factors involved in drought induction. In the analysis of cis-acting elements of *MsAHLs* in *M. sieversii*, a variety of plant hormone response elements, such as abscisic acid, auxin, and gibberellin, were also found. It is thus speculated that *MsAHLs* not only actively respond to a variety of abiotic stresses, but also enhance their stress resistance by regulating the synthesis of related plant hormones.

The expression pattern analysis of six genes revealed that most of the six *AHL* genes were induced under different stress conditions. In addition, the *MsAHL13* in this study was significantly induced in all four treatments and was selected as a candidate gene for molecular function analysis. This protein is not only located in the nucleus, but also interacts with the MsMYB1 protein. In *Arabidopsis*, overexpression of *AHL26* results in inhibition of hypocotyl elongation and delayed flowering [44]. After overexpression of *CsAHL20* in *Camellia sinensis*, the key gene *CSANR* in the biosynthesis of Epi-Catechins was significantly downregulated, and the content of Epi-Catechin was significantly reduced [45]. Conversely, after silencing of *CsAHL20*, *CSANR* was upregulated and the content of Epi-Catechin was significantly increased. *CSAHL20* inhibited the transcription of *CSANR*, thereby negatively regulating the biosynthesis of Epi-Catechins under drought. In *Poncirus trifoliata* L., two nuclear-localized AHL proteins containing AT-Hook motifs, PtrAHL14 and PtrAHL17, were identified as upstream transcriptional activators of PtrA/NINV7, interacting with A/T-rich motifs. PtrAHL14 and PtrAHL17 play positive roles in cold tolerance by regulating PtrA/NINV7-mediated Suc decomposition metabolism [28]. As indicated in Figure 7, although positive and negative controls were not included, the interaction between MsAHL13 and MsMYB1 will be further validated through complementary assays, including co-immunoprecipitation (Co-IP), bimolecular fluorescence complementation (BiFC), and microscale thermophoresis (MST). The MsAHL13 in this study also may be involved in cold damage and defense against plant pathogens, and may be an important regulatory transcription factor in *M. sieversii*.

## 4. Material and Methods

### 4.1. Identification of MsAHL Gene Family Members in M. sieversii

First, download the genome information of *M. sieversii* from Ensembl Plants (http://plants.ensembl.org/), including Genome, GFF, CDS, and protein files. Download the genome data of *Arabidopsis thaliana*, tomato (*Solanum lycopersicum* L.), and *M. sieversii* through Phytozome (https://phytozome-next.jgi.doe.gov/, accessed on 5 May 2025). Using the protein sequences of the AHL family members in *Arabidopsis thaliana* as templates, conduct a homology search of the whole genome protein sequences of *M. sieversii* using the TBtools (v2.056) software [46]. After removing redundant sequences, obtain the candidate AHL family protein sequences of *M. sieversii*. Screen the sequences through DNAMAN alignment to avoid duplication. Analyze the conserved domains in the protein sequences using the online tool CDD (conserved domain database) of NCBI (https://www.ncbi.nlm.nih.gov/Structure/bwrpsb/bwrpsb.cgi, accessed on 21 May 2025) [47]. Eliminate the protein sequences without the RGRP conserved domain, and upload the screened sequences to PFAM (http://pfam.xfam.org/, accessed on 5 May 2025) and SMART (https://smart.embl.de/, accessed on 5 May 2025) for further verification of their domains to obtain the members of the AHL gene family in *M. sieversii*.

### 4.2. Phylogenetic Analysis of the AHLs in M. sieversii

Using ClustalW integrated in MEGAX [48] (https://www.megasoftware.net/, accessed on 5 June 2025), multiple sequence alignment was performed. A phylogenetic tree was constructed via the Neighbor-Joining (NJ) algorithm with 25 *M. sieversii* AHL family members and 29 *Arabidopsis thaliana* AHL family members. The bootstrap parameter was set to 1000 replicates for validation.

### 4.3. Chromosomal Localization of MsAHLs

Based on the retrieved genomic information of AHL motifs in *M. sieversii*, we determined the chromosomal positions of all AHL motif genes and performed chromosomal localization analysis of *MsAHLs* using the genomic data of *M. sieversii*.

### 4.4. Analysis of Gene Structure, Conserved Motifs, and Phylogenetics of MsAHLs

This study utilized GSDS2.0 (http://gsds.cbi.pku.edu.cn/) online software to screen and analyze the gene structure of the AHL gene family in *M. sieversii*. MEME software (https://meme-suite.org/meme/, accessed on 10 July 2025) was used for the exploration of the conserved motifs of AHL family members. The protein sequence of *Arabidopsis thaliana* was aligned with the CDS database of *M. sieversii* and *AHL* gene sequence information obtained through a homology search. The *AHL* gene sequences of *M. sieversii* and *Arabidopsis thaliana* were combined using MEGAX and ITOL (Interactive Tree Of Life) [49] (https://itol.embl.de/, accessed on 10 July 2025), building an evolutionary tree for online software systems. The construction process adopts the Maximum Likelihood method and sets the bootstrap value to 1000.

### 4.5. Cis-Acting Element Analysis

Extract the promoter sequences of *MsAHLs* from the *M. sieversii* genome database (the nucleotide sequence upstream of the start codon by 2000 bp), and use PlantCARE [50] (http://bioinformatics.psb.ugent.be/webtools/plantcare/html, accessed on 10 July 2025) for prediction, Excel 16.0 for data analysis, and TBtools (v2.056) software for visual analysis of cis-acting components.

### 4.6. Subcellular Localization

Firstly, the specific homologous recombinant primers were designed according to the CDS of *MsAHL13* in *M. sieversii* for PCR amplification, and then the PCR products were identified and purified by gel electrophoresis. The target gene was ligated with the digested empty vector pCAMBIA-1302-GFP at 37 °C for 30 min using Exnase Ⅱ homologous recombinant enzyme. The conjugated product was mixed with the competent trans5α of *Escherichia coli*, reacted in a water bath at 42 °C for 1 min by heat shock transformation, and then recovered for 1–2 h at 37 °C and 200 r·min^−1^. After that, it was evenly coated on a solid LB plate containing 50 ng·mL^−1^ kanamycin for overnight culture. After PCR and sequencing verification, the positive plasmid was transformed into *Agrobacterium tumefaciens* GV3101 by electric shock. The positive clone was stored in 50% sterilized glycerol for use. Finally, *Agrobacterium tumefaciens* solution was transferred into tobacco leaves by tobacco leaf back injection method for transient expression. After 2–3 days of culture at room temperature, the GFP emission wavelength of 480–520 nm was observed by laser confocal microscope (Nikon A1, Tokyo, Japan) under 488 nm excitation light.

### 4.7. Real-Time Fluorescence Quantitative (qRT-PCR) Analysis

Use 4–6-week-old ‘Gala’ apple seedlings with intact leaves and roots, with at least 6 seedlings per group, repeated in 3 independent experiments. Maintain under a 16 h light/8 h dark photoperiod and a temperature regime of 25 °C (day)/18 °C (night). Sampling should be conducted at three different stress (200 mM NaCl simulated salt stress; 4 °C simulated low temperature; PEG6000 (15%) simulated drought treatment) treatments, at time points 0 h, 3 h, 6 h, 12 h, 24 h, and 48 h, with at least 3 biological replicates per time point. Healthy, disease-free mature leaves from the middle canopy of *Malus domestica* ‘Gala’ were surface-sterilized with 75% ethanol (30 s), rinsed thrice with sterile water, and air-dried. *Valsa mail* strain ACCC 30173 was cultured on Potato Dextrose Agar (Medium) at 25 °C in darkness for 5–7 days, and spore suspensions were adjusted to 1 × 10^5^–10^6^ spores/mL (hemocytometer). Post-inoculation sampling occurred at 0 h, 3 h, 6 h, 12 h, 24 h, and 48 h, with ≥3 biological replicates per time point [51,52,53]. The above materials were quickly frozen with liquid nitrogen and stored in the refrigerator at −80 °C for standby. Primer3.0 software was used to design specific primers for these genes, and Bioengineering (Shanghai, China) Co., Ltd. was entrusted to synthesize them. *Actin* was selected as the internal reference gene. A total of 60 samples (4 treatments × 5 time points × 3 biological replicates) were processed for RNA extraction. RNA was extracted from the samples by using the Huayueyang RNA plant Mini Kit (Beijing, China), and cDNA was synthesized by using the primescript RT reagentkit (Takara, Dalian, China). qRT-PCR was performed with 3 replicates using SYBR Green Master Mix (Vazyme, Nanjing, China). All experiments were conducted following the standard protocols. The qRT-PCR was performed on the StepOne Real-Time PCR System (ABI, Los Angeles, CA, USA). The qRT-PCR program was set as follows: pre denaturation at 95 °C for 30 s; then, 40 cycles were carried out, each cycle including denaturation at 95 °C for 5 s, annealing at 60 °C for 30 s and extension at 95 °C for 5 s; the last 60 °C 60 s was used for data acquisition, and the program was ended at 50 °C 30 s. The relative expression of genes was calculated by the 2^−ΔΔCT^ method [54], and the differences were analyzed by SPSS v26.0 software. All primers used for qRT-PCR can be found in Table S2.

### 4.8. Yeast Two-Hybrid Assay

We made improvements based on the methods of our predecessors [55]. Using the cDNA template of *M. sieversii*, the *MsAHL13* and *MsMYB1* were cloned separately. MsMYB1 and MsAHL13 were constructed onto the yeast two-hybrid pGBKT7 (BD) and pGAKT7 (AD) plasmids, respectively, to form fusion expression vectors. BD-MsMYB1 and AD-MsAHL13, as well as BD-empty and AD-empty, were separately transformed into yeast competent cells and plated onto SD/-Trp/-Leu double-deficient yeast medium plates. The plates were incubated upside down in a 30 °C incubator for 4 to 5 days. Six single colonies were selected and plated onto SD/-Trp-Leu-His triple-deficient medium plates, which were then incubated upside down for 3 to 5 days.

### 4.9. Luciferase Assay

Using cDNA templates from *M. sieversii*, *MsAHL13* and *MsMYB1* genes were cloned, respectively, and constructed into *pCAMBIA1300-nLUC* and *pCAMBIA1300-cLUC* vectors to form fusion expression vectors. The Agrobacterium transient expression system was used to transiently express the proteins of interest in *Nicotiana benthamiana* leaves. The assay substrate was prepared by dissolving 25 mg of D-luciferin potassium salt in 0.7852 mL of sterile water to make a stock solution of 100 mmol. Before use, the stock solution was diluted with sterile water containing 0.1% Triton-X-100 to a final concentration of 1–5 mmol. The diluted substrate was evenly applied to the infiltrated leaves, followed by a 5 min dark treatment and imaging using a plant in vivo imager.

## 5. Conclusions

In this research, a total of 25 AHL protein sequences were successfully identified from the genome of *Malus sieversii*. Remarkably, these proteins exhibited notable disparities in their physicochemical properties. The construction of a phylogenetic tree revealed that the AHL proteins of Xinjiang’s *Malus sieversii* were more evolutionarily conserved in comparison to those of cultivated apple varieties. Regarding the gene structure, the majority of *MsAHL* genes boasted a stable configuration, featuring one to four exons and one to three introns. Moreover, the AHL proteins were highly conserved, with every single one containing the PPC domain. The *MsAHL* gene family was present in the genome in a single-copy distribution pattern and was unevenly situated across 10 chromosomes. Further analysis of the promoter region of the *MsAHLs* uncovered the presence of 31 distinct types of cis-acting elements. These elements were primarily associated with light-responsiveness, plant hormone-responsiveness, abiotic stress-responsiveness, and development-related functions. In terms of evolutionary trends, when compared to model plants, the *MsAHLs* showed a greater evolutionary inclination towards tomatoes. When examined in relation to closely related species, the highest number of collinear gene pairs was found with the ‘Golden Delicious’ apple, which implies a more conservative evolutionary process. Under various abiotic stress conditions and fungal infections, six *MsAHL* genes demonstrated different levels of responses. Finally, subcellular localization experiments indicated that MsAHL13 was located in the nucleus, and it was discovered through interaction assays that MsAHL13 had a physical interaction with MsMYB1.

## Data Availability

The original contributions presented in this study are included in the article and Supplementary Material. Further inquiries can be directed to the corresponding authors.

## Supplementary Materials

The following supporting information can be downloaded at https://www.mdpi.com/article/10.3390/plants14172625/s1. Figure S1. Chromosome localization of the *MsAHL* family genes in *M. sieversii.* Table S1. Information on the *MsAHL* protein family. Table S2. Primers used in this study.
