# Supplementary figures and images for: Genome-Wide Analysis of the AT-Hook Gene Family in Malus sieversii and Functional Characterization of MsAHL13
Source: Plants (Basel). 2025 Aug 23;14(17):2625. doi: 10.3390/plants14172625 (PMC12430185; doi:10.3390/plants14172625)

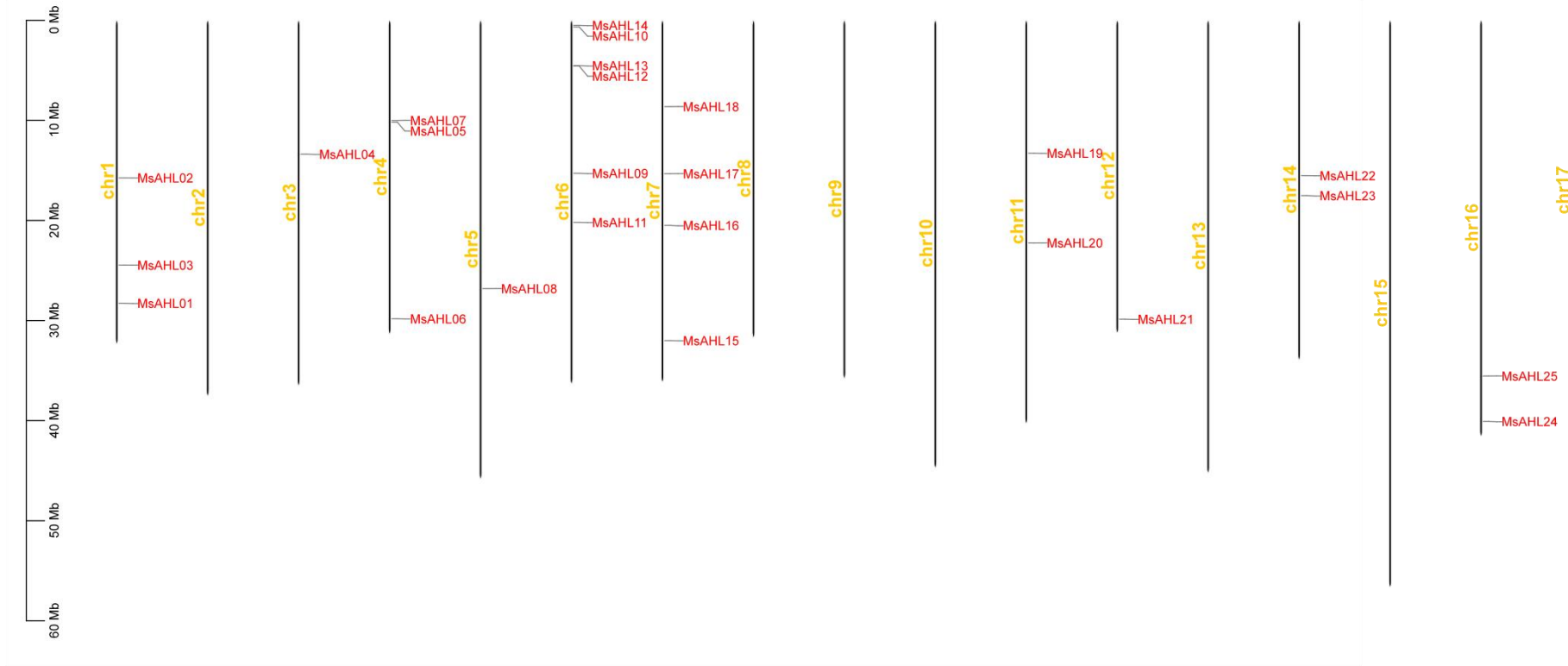

FigureS1 Chromosome localization of the MsAHL family genes in *M. sieversii*

Supplement: Supplementary file 1 [file plants-14-02625-s001.zip › FigureS1.pdf]
